# RETRACTED: Batool et al. Development and Evaluation of Cellulose Derivative and Pectin Based Swellable pH Responsive Hydrogel Network for Controlled Delivery of Cytarabine. *Gels* 2023, *9*, 60

**DOI:** 10.3390/gels11120943

**Published:** 2025-11-24

**Authors:** Nighat Batool, Rai Muhammad Sarfraz, Asif Mahmood, Umaira Rehman, Muhammad Zaman, Shehla Akbar, Diena M. Almasri, Heba A. Gad

**Affiliations:** 1 Department of Pharmaceutical Sciences, Pak-Austria Fachhochschule Institute of Applied Sciences and Technology, Haripur 22621, Pakistan; 2 Department of Pharmaceutics, Faculty of Pharmacy, University of Sargodha, Sargodha 40100, Pakistan; 3 Department of Pharmacy, University of Chakwal, Chakwal 48800, Pakistan; 4 Faculty of Pharmacy, University of Central Punjab, Lahore 54000, Pakistan; 5 College of Pharmacy, Allama Iqbal Campus, University of the Punjab, Lahore 54000, Pakistan; 6 Department of Pharmacognosy, Faculty of Pharmacy, The Islamia University of Bahawalpur, Bahawalpur 63100, Pakistan; 7 Department of Pharmacy Practice, Faculty of Pharmacy, King Abdulaziz University, Jeddah 21589, Saudi Arabia; 8 Department of Pharmaceutics and Industrial Pharmacy, Faculty of Pharmacy, Ain Shams University, Cairo 11566, Egypt; 9 Department of Pharmaceutical Sciences, Pharmacy Program, Batterjee Medical College, P.O. Box 6231, Jeddah 21442, Saudi Arabia

The journal retracts the article “Development and Evaluation of Cellulose Derivative and Pectin Based Swellable pH Responsive Hydrogel Network for Controlled Delivery of Cytarabine” [1] cited above.

Following publication, concerns were brought to the attention of the Editorial Office regarding the duplication of Figure 13 between this publication [1] and two other articles published by a similar authorship group [2,3].

Adhering to our complaint procedure, an investigation was conducted by the Editorial Office and Editorial Board that confirmed the reuse of a figure across three articles [1,2,3], each presenting the same control group image but with differing reported values. While the authors cooperated with the Editorial Office during the investigation, they were unable to provide a satisfactory explanation for the figure overlap or appropriate raw data for the Editorial Board’s evaluation. Consequently, the Editorial Board has lost confidence in the reliability of the findings and decided to retract this publication [1], as per MDPI’s retraction policy (https://www.mdpi.com/ethics#_bookmark30).

This retraction was approved by the Editor-in-Chief of the journal *Gels.*

The authors did not provide a comment on this decision.
